# The Arp2/3 complex, UNC-115/abLIM, and UNC-34/Enabled regulate axon guidance and growth cone filopodia formation in *Caenorhabditis elegans*

**DOI:** 10.1186/1749-8104-4-38

**Published:** 2009-10-02

**Authors:** Adam D Norris, Jamie O Dyer, Erik A Lundquist

**Affiliations:** 1 Department of Molecular Biosciences, University of Kansas, 1200 Sunnyside Avenue, 5049 Haworth Hall, Lawrence, KS 66045, USA

## Abstract

**Background:**

While many molecules involved in axon guidance have been identified, the cellular and molecular mechanisms by which these molecules regulate growth cone morphology during axon outgrowth remain to be elucidated. The actin cytoskeleton of the growth cone underlies the formation of lamellipodia and filopodia that control growth cone outgrowth and guidance. The role of the Arp2/3 complex in growth cone filopodia formation has been controversial, and other mechanisms of growth cone filopodia formation remain to be described.

**Results:**

Here we show that mutations in genes encoding the Arp2/3 complex (*arx *genes) caused defects in axon guidance. Analysis of developing growth cones *in vivo *showed that *arx *mutants displayed defects in filopodia and reduced growth cone size. Time-lapse analysis of growth cones in living animals indicated that *arx *mutants affected the rate of growth cone filopodia formation but not filopodia stability or length. Two other actin modulatory proteins, UNC-115/abLIM and UNC-34/Enabled, that had been shown previously to affect axon guidance had overlapping roles with Arp2/3 in axon guidance and also affected the rate of filopodia initiation but not stability or length.

**Conclusion:**

Our results indicate that the Arp2/3 complex is required cell-autonomously for axon guidance and growth cone filopodia initiation. Furthermore, they show that two other actin-binding proteins, UNC-115/abLIM and UNC-34/Enabled, also control growth cone filopodia formation, possibly in parallel to Arp2/3. These studies indicate that, *in vivo*, multiple actin modulatory pathways including the Arp2/3 complex contribute to growth cone filopodia formation during growth cone outgrowth.

## Background

The growth cone of a developing axon senses and responds to extracellular cues, resulting in the migration of the growth cone and thus axon to its correct target region in the nervous system [1,2]. Growth cones display dynamic, actin-based lamellipodial protrusions ringed by filopodia that together guide the growth cone to its target [3-5]. The Arp2/3 complex is a seven-member protein complex that nucleates actin filaments from the sides of pre-existing actin filaments. In cultured cells, the Arp2/3 complex is necessary to form the network of branched actin filaments underlying lamellipodia [6-8].

The Arp2/3 complex has been implicated in axon pathfinding *in vivo*. In *Drosophila*, the Arp2/3 complex and its regulators WAVE/Scar and Kette are required for proper axon pathfinding [9], and in *Caenorhabditis elegans*, the Arp2/3 regulators WAVE-1/WAVE, WASP-1/WASP, GEX-2/Sra-1, and GEX-3/Kette are required for proper axon guidance [10]. However, in these studies, the effects of Arp2/3 on growth cone outgrowth were not described. The role of the Arp2/3 complex in growth cone morphology has been controversial. In two studies using cultured hippocampal neurons, inhibition of Arp2/3 activity resulted in increased axon length with little or no effect on growth cone filopodia or morphology [11,12]. Another recent study using cultured hippocampal neurons and neuroblastoma cells showed that Arp2/3 knock-down resulted in defective actin organization and defective lamellipodia and filopodia formation in the growth cone [13]. Studies reported here show that Arp2/3 is indeed required for growth cone morphology and filopodia formation *in vivo*, and that Arp2/3 is required specifically for the rate of filopodia initiation but not filopodial stability or length. Thus, our studies demonstrate that Arp2/3 is required for growth cone filopodia formation and extend these findings *in vivo*, explaining the axon pathfinding defects observed in Arp2/3 mutants. Furthermore, UNC-115/abLIM has been implicated in axon pathfinding in *C. elegans *[14-16] and *Drosophila *[17], but its role in growth cone dynamics and morphology during outgrowth is not understood. Our results here show that UNC-115/abLIM is also involved in the initiation of growth cone filopodia.

Experiments described here in *C. elegans *show that mutations in genes encoding subunits of the Arp2/3 complex (*arx *genes) cause defects in axon guidance, and that Arp2/3 is required cell-autonomously in neurons for proper axon guidance. Analysis of growth cones during outgrowth *in vivo *revealed that *arx *mutants displayed reduced numbers of growth cone filopodia and also reduced growth cone size. Time-lapse analysis of growth cones in living animals indicated that the Arp2/3 complex controlled the rate of growth cone filopodia initiation but not filopodia stability or length. These results indicate that, in *C. elegans*, the Arp2/3 complex is a key regulator of growth cone filopodia initiation during outgrowth.

The actin modulatory proteins UNC-34/Enabled and UNC-115/abLIM have been shown previously to affect axon guidance in *C. elegans *[15,16,18]. Enabled is a key regulator of filopodia formation in many systems, including neurons, and is thought to act by blocking actin-capping activity, thus allowing for long filament growth in filopodia [19,20], although recent studies indicate that Enabled might have anti-capping independent roles [21] that are involved in filopodia formation [22].

The actin binding protein UNC-115/abLIM controls axon pathfinding and lamellipodia and filopodia formation in cultured cells and in *C. elegans *neurons [14,15,23]. We show here that *unc-115 *also caused defects in growth cone filopodia initiation and growth cone size, similar to *arx*.

The role of Enabled in growth cone filopodia formation has been described previously, and we found that UNC-34/Enabled affected growth cone filopodia initiation, but did not affect filopodial growth or stability once a filopodium formed. This is in contrast to previous studies that indicated that Enabled also affected filopodial elongation [19]. In sum, our results indicate that three distinct actin modulatory molecules, Arp2/3, UNC-115/abLIM, and UNC-34/Enabled, control filopodia initiation in growth cones *in vivo *that are required for proper axon guidance

## Materials and methods

### Genetic methods

All experiments were performed at 20°C using standard *C. elegans *techniques [24]. The following mutations and transgenic constructs were used: X: *unc-115(mn481 *and *ky275)*, *lqIs2 [osm-6::gfp]*, *mig-2(mu28)*; I: *arx-7(ok1118)*, *hT2 [bli-4(e937) let-?(q782) qIs48] (I;III)*, *lqIs40 [gcy-32::gfp]; *II: *juIs76 [unc-25::gfp]*; III: *arx-4(ok1093)*, *sC1 [dpy-1(s2170)]*; IV: *ced-10(n1993)*, *lqIs3 [osm-6::gfp]*, *nT1 [qIs51] (IV;V); V: arx-2(ok1269)*, *unc-34(e951)*. The chromosomal locations of *lqIs49 [gcy-32::gfp] *and *lqIs75 [gcy-32::arx-7::mCherry] *were not determined. The *arx-2(+)*, *arx-4(+)*, and *arx-7(+) *whole-gene regions were maintained as extrachromosomal arrays. Extrachromosomal arrays were generated by germ line microinjection and integrated into the genome by standard techniques [25].

*arx-2*, *arx-4*, and *arx-7 *mutations were balanced by the rearrangements *nT1*, *sC1*, and *hT2*, respectively. *nT1 *and *hT2 *harbored transgenes that drove *gfp *expression in the pharynx, whereas *sC1 *had no *gfp *marker. *arx-2 *and *arx-7 *homozygotes were identified by lack of pharyngeal green fluorescent protein (GFP), whereas *arx-4 *homozygotes were identified by the protruding vulva (Pvl) phenotype in young adults. *arx-4 *L1 larvae were not imaged because they could not be unambiguously identified as could *arx-2 *and *arx-7*. Double mutants with *ced-10 *were confirmed by the gonad misrouting phenotype and by the persistent cell corpse phenotype [26], and double mutants with *mig-2 *were confirmed by the gonad misrouting phenotype [27]. Double mutants with *unc-34 *and *unc-115 *were confirmed by the uncoordinated phenotype of these mutants.

### *arx *transgene construction

**Figure 1:**
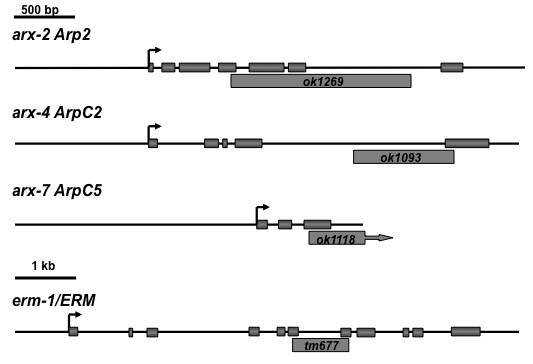
**Mutations in *arx-2*, *arx-4*, *arx-7*, and *erm-1***. The structures of the *arx-2*, *arx-4*, *arx-7*, and *erm-1 *genes are shown. Filled boxes represent exons, arrows represent translational start sites in putative cDNAs, and 5' is to the left. The extent of deletions in each of the genes is shown below the structure. The *arx-7(ok1118) *deletion extends into the gene to the 3' of *arx-7*.

The *arx-2*, *arx-4*, and *arx-7 *genes were amplified by PCR from wild-type N2 genomic DNA (Figure 1). The coding regions and splice junctions were sequenced to ensure that no mutations were introduced by PCR. The sequences of all primers and amplified regions used in this work are available upon request.

For cell-specific rescue, the coding regions of *arx-4 *and *arx-7 *(from the initiator methionine to the stop codon) were amplified from N2 genomic DNA and placed behind the *osm-6 *promoter to drive PDE expression, or the *gcy-32 *promoter to drive PQR expression. For *arx-7*, the stop codon was not included, and the coding region was fused in frame to the coding region for GFP.

### Analysis of PDE axon guidance defects

As previously described, PDE axons were visualized with an *osm-6 promoter::gfp *transgene [15]. *osm-6::gfp*, expressed in all ciliated sensory neurons including PDE, allows unambiguous identification of the PDE axon from other axons [28]. A PDE axon was considered defective if the axon failed to reach the ventral nerve cord or if it reached the ventral nerve cord at a position greater than a 45° angle from the PDE cell body. Young adult animals were scored, although *arx *mutants were often scored as L3 or L4 larvae. Percentages of defective PDE axon guidance in each strain were determined, and significance was judged by a two-sided *t*-test with unequal variance unless otherwise noted.

### Imaging and analysis of PQR dendrite development

The PQR dendrite was visualized using *gcy-32::gfp *transgenes [29]. Larvae were synchronized by washing adults and larvae from a plate with many eggs and allowing the eggs to hatch. Newly hatched larvae were washed from the plate at 0.5 h intervals and allowed to develop for the following times before imaging: 6 h, 6.5 h, 7 h, 7.5 h, and 8 h. Animals were imaged using a Leica DMR microscope with a Qimaging Retiga EXi camera. Z-stacks of each PQR were captured and were subject to nearest neighbor deconvolution using Openlab software (Improvision, Waltham, MA USA).

Filopodia were defined as growth cone protrusions that were less than 0.5 μm in width. The number of filopodial protrusions from the growth cone of PQR neurons at the 7 to 7.5 h timepoint was determined by scrolling through each Z-stack of images. An average number and standard deviation of filopodial protrusions per PQR dendrite was determined, and significance of difference between genotypes was determined by a two-sided *t*-test with unequal variance unless otherwise noted.

The lamellipodial perimeter of the growth cone was used as an indicator of growth cone size. At the 7 to 7.5 h timepoint, the distal 3 μm of the tip of the PQR dendritic protrusion was traced using ImageJ software, and a value representing the perimeter of this region was derived. An average was derived from multiple tracings, and significance of difference between genotypes was determined by a two-sided *t*-test with unequal variance unless otherwise noted.

### Time lapse imaging of VD growth cones

VD growth cones were imaged using an *unc-25::gfp *transgene *juIs76 *[30]. Early L2 stage larvae were isolated by washing hatched worms off of a plate and allowing eggs to hatch for one-half hour. Newly hatched larvae were washed off and allowed to develop at 20°C for 16 h. Larvae with visible VD growth cones were imaged. Animals were transferred to a 2% agarose pad with a drop of 10 mM muscimol (Sigma-Aldrich, St. Louis, MO USA) in M9 [31]. The drop was allowed to evaporate for 4 to 5 minutes before a coverslip was placed over the animals. Growth cones that were just emerging from the ventral muscle quadrant were imaged with a Leica DMR microscope with a BD CARV II wide-field light source and QImaging Rolera mGi camera at intervals of 120 s. Total duration of time-lapse imaging ranged from 20 to 60 minutes.

Dynamic projections emanating from the growth cone less than 0.5 μm in width were scored as filopodia. Filopodia length was measured using ImageJ software. Growth cone translocation speed was determined by measuring the distance from the distal tip of the lamellipodial portion of the growth cone to a fixed reference point along the axon, often a point at which the axon has crossed a lateral axon tract, which can cause a slight bulge in the axon. Significance of difference was determined by a two-sided *t*-test with unequal variance.

## Results

### Mutations in *arx-2*, *arx-4*, and *arx-7 *cause PDE axon guidance defects

The Arp2/3 complex is composed of seven molecules, Arp2, Arp3, and ArpC1-ArpC5 [8]. The *C. elegans *genome contains genes encoding each of these subunits. Deletion mutations in genes encoding three subunits were analyzed; *arx-2 Arp2*, *arx-4 ArpC2*, and *arx-7 ArpC5 *(Figure 1). *arx-2(ok1269) *was a 1,330-bp deletion that removed part of exon 4 and all of exons 5 and 6. *arx-4(ok1093) *was a 772-bp deletion that removed the 3' splice site of intron 4 and part of exon 5. *arx-7(ok1118) *was a 1,352-bp deletion that removed most of exon 3 of *arx-7 *(almost half of the *arx-7 *coding region) and extended into the downstream gene *Y54E10BR.1*, removing exon 1 and most of intron 1. While *arx-2(ok1269) *and *arx-4(ok1093) *are predicted to affect only *arx-2 *and *arx-4*, respectively, *arx-7(ok1118) *might affect both *arx-7 *and *Y54E10BR.1*.

From mothers heterozygous for each mutation (wild-type maternal contribution, M+), *arx-2(ok1269M+)*, *arx-4(ok1093M+)*, and *arx-7(ok1118M+) *homozygotes grew slowly and arrested as larvae or young adults. Animals that survived to adulthood were sterile and had protruding vulvae (Pvl phenotype). Most *arx-2(ok1269M+) *animals arrested before reaching adulthood in the L3 or L4 larval stage. *arx-7(ok1118) *animals arrested in L4 or early adulthood, and *arx-4(ok1093) *animals survived to adulthood but were sterile and Pvl. RNA interference of *arx-2 *and *arx-7 *caused embryonic lethality due to defects in gastrulation as previously described [32], indicating that *arx-2*, *arx-7*, and likely *arx-4 *have strong maternal contributions that are sufficient for embryonic and larval development. The variation in arrest phenotype of *arx *mutants could be due to differences in maternal contribution and not distinct functions of each *arx *gene.

**Figure 2:**
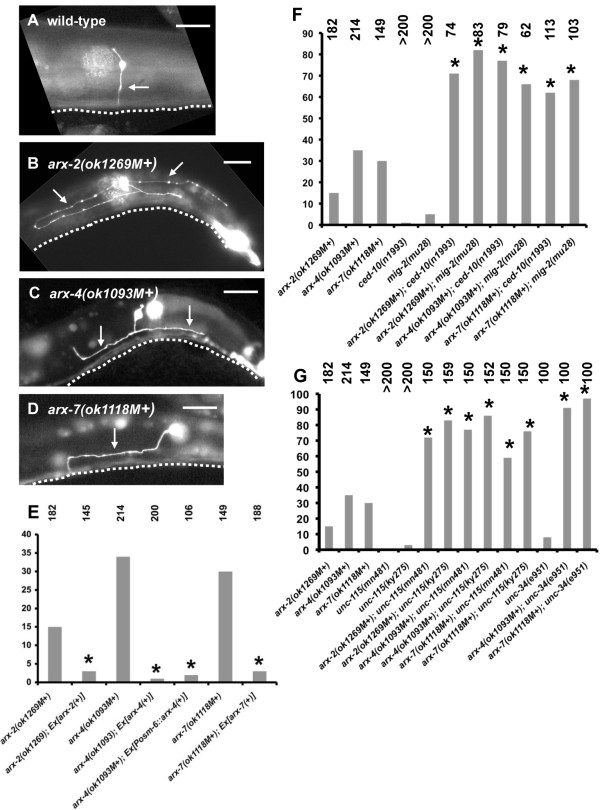
**PDE axon pathfinding defects in *arx *mutants**. **(A-D) **Shown are fluorescent micrographs of larval or young adult animals with *osm-6::gfp *expression in the PDE neurons. (A) A wild-type PDE neuron extended a single unbranched axon (arrow) straight ventrally to the ventral nerve cord (VNC), which is out of focus but represented by a dashed line. (B-D) PDE axons were misguided in *arx-2*, *arx-4*, and *arx-7 *mutants. The axons failed to reach the VNC and wandered laterally (B, C) or wandered laterally before extending to the VNC (D). In all micrographs, anterior is to the left and dorsal is up; dashed lines represent the location of the out-of-focus VNC, and scale bars = 10 μm. In genotypes, 'M+' denotes that the animal had wild-type maternal *arx *contribution. **(E-G) **Quantification of PDE axon guidance defects. Genotypes are along the x-axis, and proportion of misguided PDE axons is on the y-axis. The numbers of PDE axons scored for each genotype is listed above the graph. In (E), *Ex [arx(+)] *indicates that the animals harbored a wild-type *arx(+) *transgene, and *Ex [Posm-6::arx-4] *indicates that the animals harbored a transgene that expressed the *arx-4(+) *gene specifically in the PDEs. The differences between *arx *mutants alone and those harboring transgenes were significant in all cases (*P *≤ 0.0001, indicated by an asterisk).

Axon morphology of the PDE neuron was scored in *arx *homozygous animals. The PDEs are bilateral sensory neurons in the posterior of the animal with single, unbranched axons that extend ventrally to the ventral nerve cord (VNC), where the axons bifurcate and extend anteriorly and posteriorly in the VNC (Figure 2A) [33]. In each *arx *homozygous mutant, defects in axon guidance were observed (Figure 2B-D). *arx-2(ok1269M+) *displayed 15% PDE axon guidance defects, *arx-4(ok1093M+) *34%, and *arx-7(ok1118M+) *30% (Figure 2E). Defects included failure of the axon to extend straight ventrally with lateral wandering, often never reaching the VNC (Figure 2B). Mutant PDE axons sometimes bifurcated prematurely before reaching the VNC (Figure 2C). Furthermore, some axons appeared to initiate from the sides of the PDE cell body rather than the ventral surface (Figure 2D). This could reflect a defect in polarity of axon initiation or could be a consequence of growth cone misguidance. *arx *mutants did not display a high frequency of ectopic axon formation as do other mutations that also affect PDE axon guidance (for example, *mig-2*, *ced-10 *and *unc-115*).

To ensure that the phenotype observed in *arx *mutants was due to disruption of the *arx *gene, the entire wild-type genomic region for each locus was amplified by PCR and used in transgenic rescue experiments (see Materials and methods). *arx-4 *is the downstream gene in a *C. elegans *operon, and the entire operon region composed of the genes *K02F3.1*, *K02F3.12*, and *Y6D11A.1 *was included in the fragment. The *arx-2(+) *and *arx-4(+) *transgenes rescued the slow growth, sterility and pVul phenotype of *arx-2(ok1269) *and *arx-4(ok1093)*, respectively, and transgenic animals were viable and fertile. Furthermore, PDE axon defects were completely rescued (Figure 2E; 15% to 3% for *arx-2(ok1269M+) *and 34% to 1% for *arx-4(ok1093)*; *P *< 0.0001 for each). The *arx-7(+) *fragment rescued the slow growth and pVul components of the *arx-7(ok1118) *phenotype as well as PDE axon pathfinding defects (30% to 3%; *P *< 0.0001; Figure 2E). However, *arx-7(ok1118); Ex [arx-7(+)] *animals were still sterile. The *arx-7(ok1118) *deletion also affected the downstream gene *Y54E10BR.1*, which was not included in the rescuing fragment. The sterility of *arx-7(ok118); Ex [arx-7(+)] *transgenic animals might be due to the effects of *ok1118 *on *Y54E10BR.1*. These data indicate that the PDE axon guidance defects observed in *arx *mutants are due to mutation of the *arx *genes.

### *arx-4 *acts cell-autonomously in PDE axon guidance

To determine if *arx-4 *acts in neurons to control axon pathfinding, a transgene was constructed consisting of the *osm-6 *promoter upstream of the *arx-4 *coding region. The *osm-6 *promoter is active exclusively in ciliated sensory neurons including PDE and has no apparent activity in other tissues [15,28]. *arx-4(ok1093M+) *animals harboring the *Posm-6::arx-4(+) *transgene were slow-growing, Pvl, and sterile, similar to *arx-4(ok1093) *homozygotes alone, confirming that the *Posm-6::arx-4(+) *transgene was not broadly active. However, PDE axon guidance defects were rescued in these animals (34% to 2%; *P *< 0.0001; Figure 2E), suggesting that expression of *arx-4 *in the PDE neurons rescued *arx-4 *axon guidance defects. These results suggest that *arx-4 *acts cell-autonomously in the PDE neurons in axon guidance.

### *arx *mutations enhance mutations in *ced-10/Rac, mig-2/RhoG*

Previous results indicated that the Rac-like molecules CED-10/Rac [26] and MIG-2/RhoG [27] act redundantly in axon guidance [34] and that the Arp2/3 activators WVE-1/WAVE and WSP-1/WASP might act in the CED-10/Rac and MIG-2/RhoG pathways, respectively [10]. The loss-of-function alleles *ced-10(n1993) *and *mig-2(mu28) *enhanced the PDE axon pathfinding defects of *arx-2(ok1269M+)*, *arx-4(ok1093M+)*, and *arx-7(ok1118M+) *(Figure 2F). For example, *ced-10(n1993); arx-4(ok1093M+) *displayed 77% PDE axon guidance defects compared to 34% for *arx-4(ok1093M+) *alone; and *mig-2(mu28); arx-7(ok1118M+) *displayed 68% compared to 30% for *arx-7(ok1118M+) *alone (*P *< 0.0001 for all comparisons). Double mutants did not exhibit ectopic axon formation as observed in *ced-10; mig-2 *double mutants [34]. That each *arx *mutation was enhanced by both *ced-10(n1993) *and *mig-2(mu28) *suggests that the Arp2/3 complex might act in both the CED-10 Rac and MIG-2 RhoG pathways in PDE axon guidance. Possibly, both WVE-1/WAVE and WSP-1/WASP pathways converge on regulation of Arp2/3 in axon guidance.

### The actin-binding proteins UNC-115/abLIM and UNC-34/Enabled have redundant function with Arp2/3 in axon guidance

Previous studies showed that the actin-binding protein UNC-115/abLIM acts downstream of Rac signaling in axon guidance [15], raising the possibility that Rac signaling employs multiple actin-modulating pathways in axon guidance (UNC-115/abLIM and Arp2/3). Indeed, *unc-115 *mutations enhanced PDE guidance defects of *arx *mutants (Figure 2G). For example, *unc-115(ky275); arx-2(ok1269M+) *displayed 83% PDE axon guidance defects compared to 15% for *arx-2(ok1269M+) *alone (*P *< 0.0001 for all differences). Double mutants did not display enhanced ectopic axon formation as observed in double mutants of *unc-115 *with *ced-10 *and *mig-2*. That *unc-115 *mutations enhanced PDE axon guidance defects of *arx *mutations indicates that UNC-115/abLIM acts in parallel to Arp2/3 in PDE axon guidance, a result consistent with the idea that Rac GTPases employ both Arp2/3 and UNC-115/abLIM.

Previous studies showed that UNC-115/abLIM and UNC-34/Enabled had redundant roles in PDE axon guidance [16]. As predicted by this result, UNC-34/Enabled and Arp2/3 also had parallel function in PDE axon guidance (Figure 2G). Alone, loss-of-function *unc-34(e951) *mutants displayed 8% PDE guidance defects. *unc-34(e951); arx-4(ok1093) *and *unc-34(e951); arx-7(ok1118M+) *displayed 91% and 97% PDE guidance defects compared to 35% and 35% for the *arx *mutants alone (*P *< 0.0001 for each comparison). Together, these data indicate that Arp2/3, UNC-115/abLIM, and UNC-34/Enabled all act redundantly to control PDE axon guidance.

**Figure 3:**
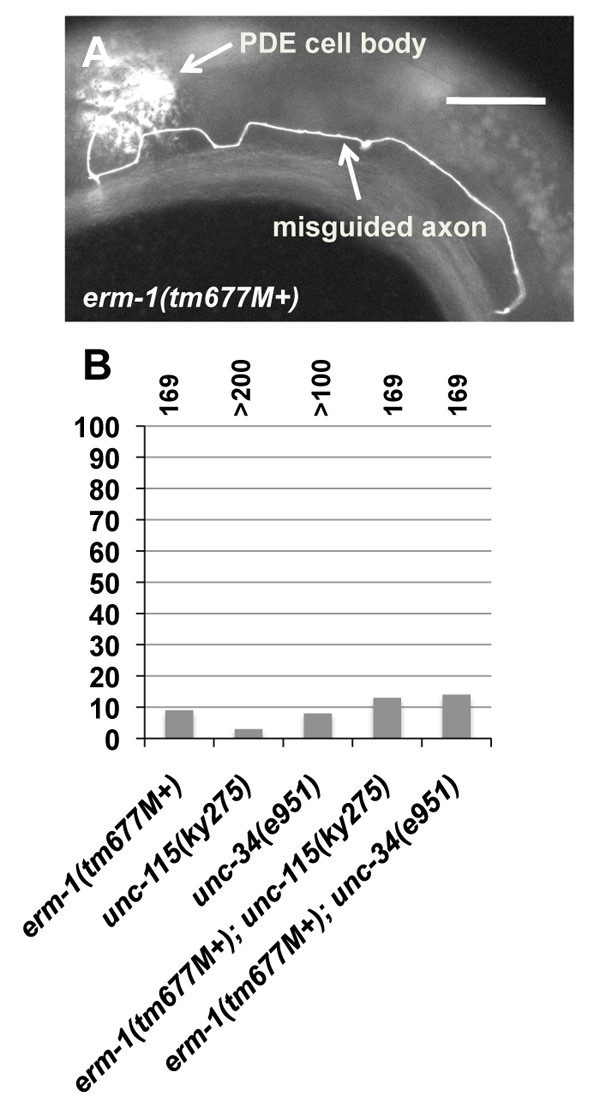
**PDE axon guidance defects in *erm-1(ok677M+) *and double mutants**. **(A) **A micrograph of a misguided PDE axon in *erm-1(tm677M+)*. The scale bar represents 10 μm. **(B) **Quantification of PDE axon guidance defects as described in Figure 2.

Ezrin/radixin/moesin (ERM) proteins are regulators of actin organization and interaction with the plasma membrane. The *C. elegans *genome encodes one molecule similar to ezrin, radixin, and moesin called ERM-1. *erm-1(tm677) *is an out-of-frame deletion of the locus (Figure 1) that causes maternal-effect lethality (that is, homozygotes from a heterozygous mother survive until adulthood). RNA interference of *erm-1 *results in disrupted morphogenesis of the gut lumen [35,36]. We found that *erm-1(tm677M+) *animals displayed weak but significant defects in PDE axon guidance (9%; Figure 3). Double mutants of *erm-1(tm677M+) *with *unc-115(ky275) *and *unc-34(e951) *displayed PDE axon guidance defects that were not significantly different from the singles alone. These results indicate that ERM-1 has a role in PDE axon guidance but does not act redundantly with UNC-115 and UNC-34. Thus, the synergistic enhancement of *arx *mutants by *unc-34 *and *unc-115 *is not likely due to general actin cytoskeletal disruption. Rather, these results suggest that the genetic interaction is specific and that Arp2/3, UNC-115, and UNC-34 might act in a common process in axon guidance not affected by ERM-1.

### Development of the posterior PQR neurite involves a growth-cone-like structure with filopodia

Mutations in *arx *genes caused axon guidance defects, but the effect of *arx *mutations on the growth cone during neurite extension was unclear. Developing neurites in *arx *mutants were analyzed. Transgenes used to assay PDE axon morphology were not visibly expressed early in PDE development during axon extension, including *osm-6::gfp *[28] and *cat-1::gfp *[37]. Therefore, the development of a distinct neurite was examined. A *gcy-32::gfp *reporter [29] was used to analyze outgrowth of the posterior PQR neurite, which develops into a ciliated sensory dendrite [33].

**Figure 4:**
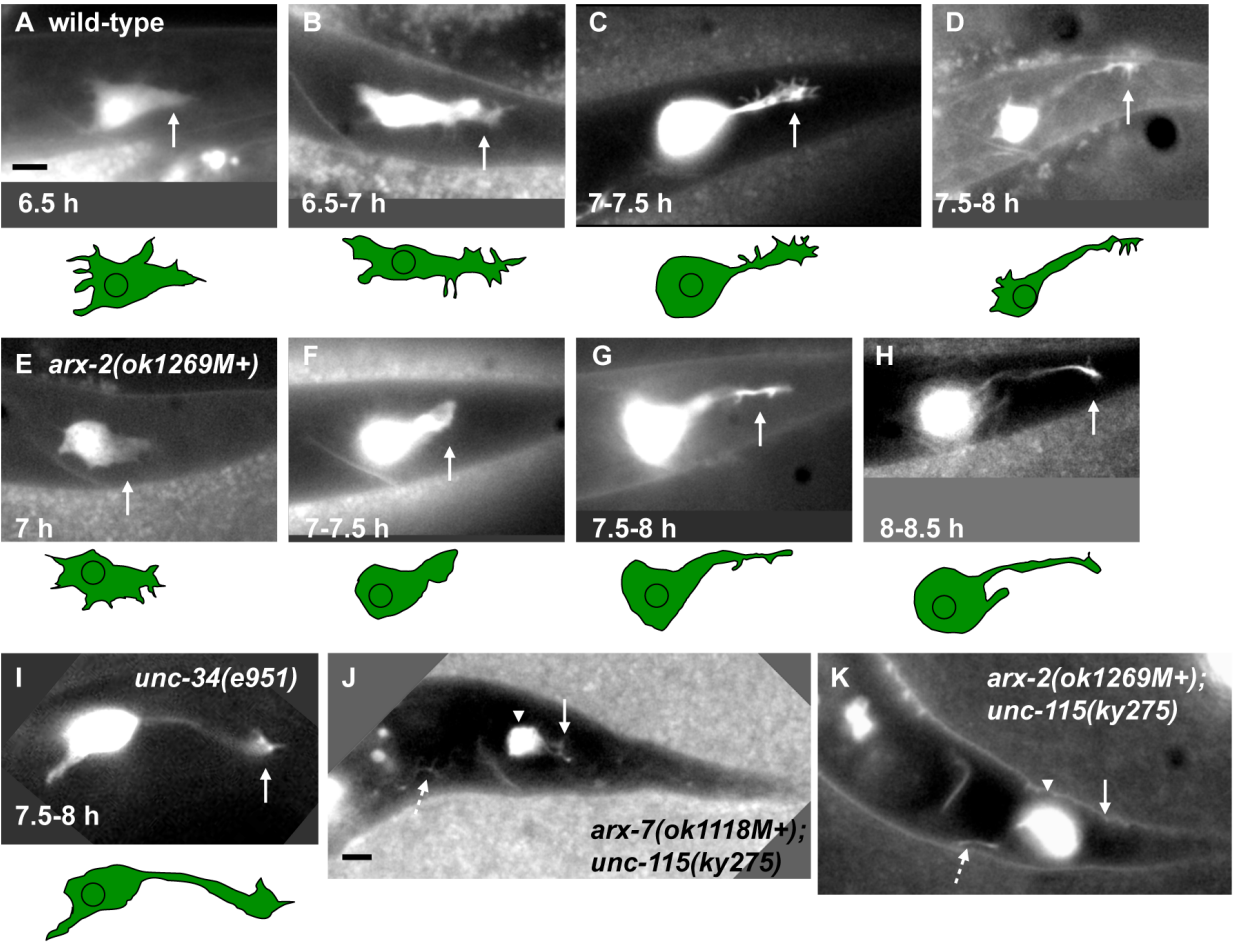
***arx *mutants display fewer PQR growth cone filopodia**. Shown are fluorescent micrographs of living animals with *gcy-32::gfp *expression in the PQR neuron in the tail of the animal posterior to the anus. Below each micrograph is a tracing showing the shape of the PQR neuron. **(A-D) **Time courses of wild-type posterior PQR neurite development (arrows). PQR neurons from different animals at particular timepoints are shown. A tracing of the cell and neurite is shown below each micrograph of animals at given timepoints after hatching (see Materials and methods). **(E-H) **PQR posterior neurite development in a representative example of an *arx-2(ok1269M+) *mutant. **(I) **The PQR posterior neurite from an *unc-34(e951) *mutant. **(J-K) **PQR neurons from *unc-115(ky275); arx-2(ok1269M+) *or *unc-115(ky275); arx-7(ok1118M+) *mutants at 9 h after hatching at a time when wild-type posterior protrusions had fully extended. The PQR in (J) showed a rudimentary posterior protrusion (arrow), and the PQR neuron in (K) showed no obvious posterior protrusion (arrow). The dashed arrows in (J) and (K) point to normal anterior axonal protrusions from these PQR neurons. Arrowheads indicate the PQR cell bodies. In all micrographs, anterior is to the left and dorsal is up, and the scale bars represent 2 μm (scale bar in (A) is for (A-I); that in (J) is for (J-K)).

**Figure 5:**
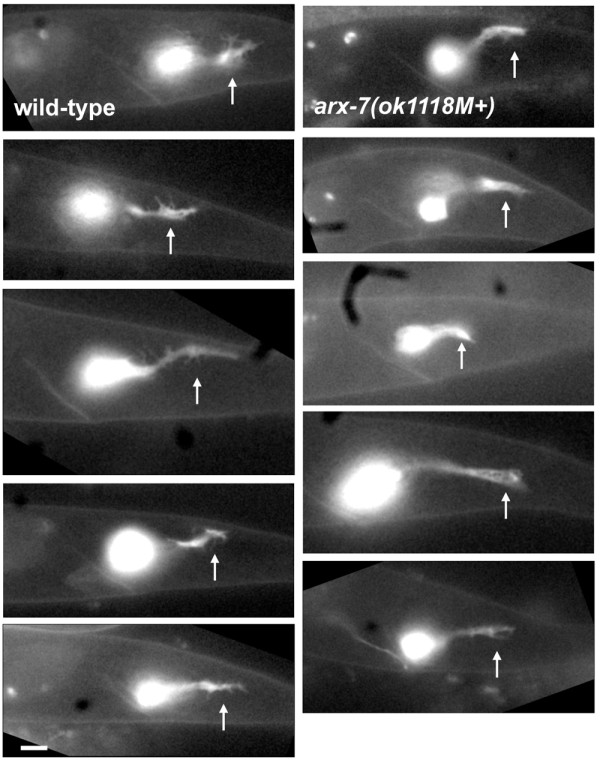
***arx-7(ok1118M+) *displayed fewer growth cone filopodia than wild type**. Fluorescent micrographs of posterior PQR neurites in representative samples of wild type (left column) and *arx-7(ok1118M+) *(right column) at 7 to 8 h after hatching. Arrows point to growth cones. For all micrographs, anterior is left, dorsal is up, and the scale bar = 2 μm.

The PQR neuron, a descendant of the QL neuroblast, is born in the posterior of the L1 larva and migrates to its final position in the tail near the phasmid ganglia [38]. At 6.5 h after hatching, the PQR cell body had reached its final position and differentiation began. At this time, *gcy-32::gfp *expression was evident, revealing a large, wedge-shaped cell body with multiple finger-like filopodial protrusions emanating from around the cell body (Figure 4A). From 6.5 to 7 h, posterior PQR protrusions were oriented to the dorsal-posterior and appeared as thickened, foot-like structures with smaller filopodial extensions (Figure 4B). From 7 to 7.5 h, posterior extensions thinned into neurite-like structures with distal growth cones and multiple filopodial protrusions (Figure 4C). Filopodia also protruded along the lengths of the neurites. Also at this time, the cell bodies of the PQR neurons began to resemble a typical neuronal cell body: they became smaller in size and oval-shaped with fewer filopodial extensions. From 7.5 to 8 h, the neurites were thin and the growth cones at their tips continued to extend (Figure 5D). Filopodial extensions became mostly restricted to the distal growth cone at this time. By 8.5 h, the neurites had extended completely and the growth cone-like structures were no longer evident. In broad terms, PQR dendritic extension involved initial dorsal-posterior protrusion, consolidation of the protrusion into a neurite with a growth cone, and growth cone extension. This is similar to growth cone advance observed in cultured neurons, which includes a period of growth cone swelling (engorgement) followed by a narrowing and consolidation of the proximal growth cone to form the new axon shaft [39].

**Figure 6:**
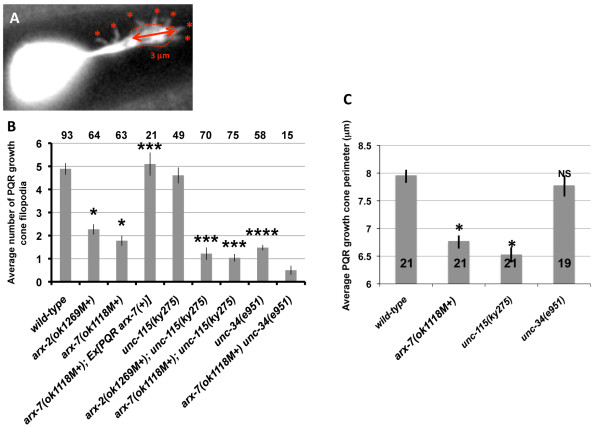
**Quantification of PQR posterior neurite growth cone filopodia defects**. **(A) **A micrograph demonstrating how filopodia number and growth cone perimeter was quantified. Asterisks mark filopodial protrusions (<0.5 μm in width). The red dashed line is a tracing of the distal 3 μm of the growth cone perimeter. **(B) **The y-axis represents the average number of filopodia present on the PQR posterior neurite growth cone at 7 to 8 h after hatching. Error bars represent standard error of the mean. *arx-2(ok1269M+) *and *arx-7(ok1118M+) *displayed significantly fewer filopodia than wild type (* *P *< 0.0001). Cell-specific expression of the *arx-7(+) *in PQR via the *Pgcy-32::arx-7(+):: gfp *transgene rescued filopodia defects of *arx-7(ok1118M+) *(** *P *< 0.0001). *unc-115(ky275) *was not significantly different from wild type (*P *= 0.51), but did significantly enhance *arx-2(ok1269M+) *and *arx-7(ok1118M+) *(*** *P *= 0.003 and 0.007, respectively). *unc-34(e951) *displayed significantly fewer filopodia than wild type (**** *P *< 0.0001). **(C) **Growth cone perimeter (in microns) is on the y-axis. Error bars represent standard error of the mean. *arx-7(ok1118M+) *and *unc-115(ky275) *had reduced growth cone perimeter compared to wild type (* *P *< 0.001) whereas *unc-34 *did not (NS = not significant).

The finger-like extensions on PQR growth cones resembled filopodia. In many systems, including cultured cells and neurons and *C. elegans *neurons [40,41], Enabled activity is required for the formation of filopodia. To confirm that the finger-like protrusions that emanated from the PQR dendrite were filopodia, we determined if they were affected by mutation in *unc-34/Enabled*. Indeed, in *unc-34(e951) *mutants at 7 to 8 h post-hatching, the average number of finger-like extensions on PQR dendrites (defined as having a thickness of 0.5 μm or less) was reduced significantly compared to wild-type (Figures 4I and 6; 4.89 compared to1.48, *P *< 0.0001). That these finger-like protrusions were affected by *unc-34/Enabled *suggests that they are *bona fide *filopodia.

### *arx *mutants affect PQR growth cone morphology and filopodia formation

Posterior PQR neurite extension was assessed in *arx-2(ok1269M+) *and *arx-7(ok1118M+) *living larvae expressing *gcy-32::gfp*. These larvae exhibited a general growth delay, and timing of PQR dendritic events were slowed by approximately one-half hour. At 7 h, initial wedge-like cell shape with filopodia appeared normal in *arx-2 *and *arx-7 *mutants (Figure 4E). From 7 to 7.5 hours, *arx-2 *and *arx-7 *extended dorsal-posterior foot-like structures. However, these protrusions displayed fewer filopodial extensions than wild type (Figure 4F). Neurite consolidation appeared normal in *arx-2 *and *arx-7*, but from 7.5 to 8.5 hours, the growth cones at the distal tip displayed fewer filopodia (Figure 4G,H). Furthermore, fewer filopodia were evident along the length of the neurites. The posterior protrusions and growth cones of *arx-2 *and *arx-7 *mutants often appeared thinner and less ramified than in wild type, possibly indicating a growth cone lamellipodial defect as well (see Figure 5 for additional representative images of wild-type and *arx-7(ok1118M+) *growth cones).

The number of filopodia on the growth cones and neurites of wild type (7-8 h), *arx-2(ok1269M+)*, and *arx-7(ok1118M+) *(7.5-8.5 h) were counted (Figure 6A; see Materials and methods). Wild type displayed an average of 4.89 filopodia per neurite whereas *arx-2(ok1269M+) *displayed 2.27 and *arx-7(ok1118M+) *1.78 (*P *< 0.0001 for each; Figure 6B).

As mentioned previously, the growth cones of *arx *mutants often appeared less robust and smaller in size than wild type. To quantify this defect, the perimeters of growth cones (excluding filopodia) from wild type and mutant backgrounds were traced (Figure 6A; see Materials and methods). The growth cone was defined as the widened region at the tip of the neurite, starting at 3 μm from the distal growth cone tip. Wild-type growth cones displayed an average perimeter of 7.96 ± 0.91 μm (Figure 6C). *arx-7 *displayed a significantly reduced average growth perimeter (6.77 ± 0.51 μm; *P *< 0.001).

**Figure 7:**
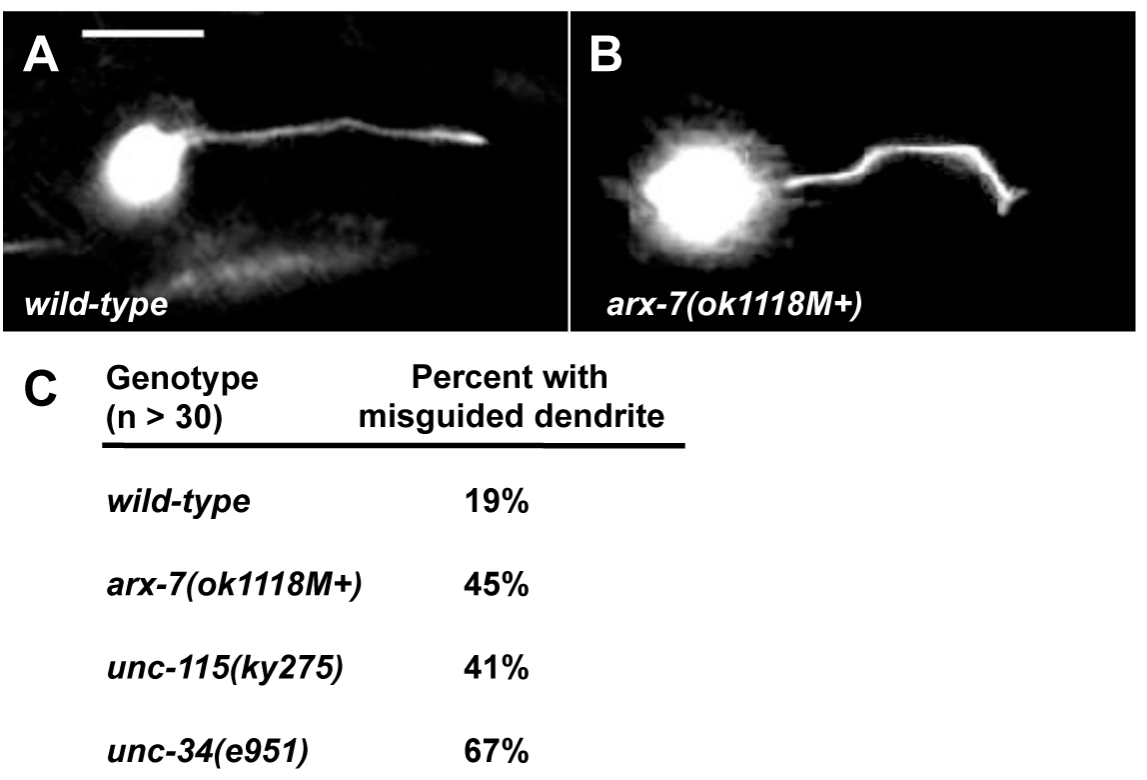
**PQR dendrite guidance defects**. **(A, B) **Micrographs of young adult PQR neurons expressing *gcy-32::gfp*. Anterior is to the left, and dorsal is up. In wild type, the PQR dendrite extends directly to the posterior. In *arx-7(ok118M+)*, the PQR dendrite extends posteriorly but has multiple turns. The scale bar in (A) represents 5 μm. **(C) **Quantification of PQR dendritic guidance defects.

To determine if these growth cone defects resulted in neurite guidance defects, we scored the final morphology of the PQR dendrites after outgrowth. Initial posterior protrusion, axon consolidation, and outgrowth of the neurite to its normal position in the tail were generally normal in *arx *mutants. However, PQR dendrites did not extend directly to the posterior as in wild type (Figure 7A). Rather, the dendrites displayed multiple turns, resulting in an undulating dendrite appearance (Figure 7B,C; 45% in *arx-7 *compared to 19% in wild type). These data indicate that *arx-7(ok1118M+) *affects the guidance of the PQR dendrite.

### *arx-7 *acts cell-autonomously in PQR filopodia regulation

A transgene consisting of the *gcy-32 *promoter driving *arx-7 *tagged with coding region for GFP was constructed. This transgene was predicted to express full-length ARX-7 with GFP at the carboxyl terminus. Animals harboring this transgene showed ARX-7::GFP fluorescence in the URX, AQR, and PQR neurons and nowhere else (data not shown). *arx-7(ok1118M+) *animals harboring this transgene displayed the gross phenotype of *arx-7(ok1118M+) *alone (arrested larvae and sterile, Pvl adults), indicating that *arx-7(+) *activity was not widely provided by the *Pgcy-32::arx-7::gfp *transgene. However, this transgene rescued filopodia formation to wild-type levels in *arx-7(ok1118M+) *PQR neurites (Figure 6B): an average of 5.1 filopodia per neurite compared to 1.78 for *arx-7(ok1118M+) *alone (*P *< 0.0001). *arx-7 *rescue was not significantly different than wild type (5.1 versus 4.89; *P *= 0.71). These data indicate that *arx-7 *acts cell-autonomously in the regulation of growth cone filopodia.

### *unc-115/abLIM *mutation enhanced PQR filopodia defects of *arx-2 *and *arx-7*

As reported above, *unc-115 *mutations enhanced PDE axon guidance defects of *arx-2*, *arx-4*, and *arx-7*, indicating redundancy of function in PDE axon guidance. The effect of *unc-115 *on PQR posterior neurite extension was examined. Alone, *unc-115(ky275)*, a null allele, displayed no defects in PQR posterior neurite filopodia formation (for example, 4.61 filopodia versus 4.89 for wild type, *P *= 0.51; Figure 6B). However, *unc-115(ky275) *enhanced filopodia defects of *arx-2(ok1269M+) *and *arx-7(ok1118M+)*. *unc-115(ky275); arx-2(ok1269M+) *displayed 1.22 filopodia per neurite compared to 2.27 for *arx-2(ok1269M+) *alone (*P *= 0.003), and *unc-115(ky275); arx-7(ok1118M+) *displayed 1.04 compared to 1.78 for *arx-7(ok1118M+) *alone (*P *= 0.007).

*unc-115(ky275) *mutants also displayed a reduced growth cone perimeter (Figure 6C; 6.52 ± 0.57 μm compared to 7.96 ± 0.91 μm for wild type; *P *< 0.001), indicating that UNC-115 might be involved in growth cone lamellipodium regulation. *unc-115(ky275) *displayed 41% PQR dendrite guidance defects (the undulating dendrite), compared to 19% for wild type (Figure 7C). Alone, *unc-115(ky275) *had no affect on filopodia, suggesting that *unc-115 *PQR dendrite guidance defects were due to affects on lamellipodia and not filopodia.

*unc-115; arx *double mutants displayed a defect not seen in *unc-115*, *arx-2*, or *arx-7 *single mutants, a rudimentary or absent posterior PQR neurite (Figure 4J,K): 12 of 70 PQR neurons from *unc-115(ky275); arx-2(ok1269M+) *and 9 of 75 *unc-115(ky275); arx-7(ok1118M+) *animals lacked a significant posterior protrusion. Some animals showed a rudimentary posterior protrusion (Figure 4J) whereas other showed no posterior neurite (Figure 4K). These observations suggest that in addition to being required for filopodia formation, the Arp2/3 complex and UNC-115/abLIM might also redundantly control neurite initiation or the ability of a neurite to form and extend.

### *unc-34/Enabled *affects filopodia but not growth cone perimeter

As previously observed in the HSN neuron [40], *unc-34(e951) *mutants displayed severely reduced filopodia-like protrusions on the PQR dendritic neurite (Figures 4I and 6; 1.48, *P *< 0.0001). That *unc-34 *affected these thin, filopodia-like protrusions supports the notion that they are *bona fide *filopodia, as Enabled molecules have been shown to control filopodia formation in many systems, including *C. elegans *neurons [40,41]. *arx-7(ok1118M+); unc-34(e951) *displayed significantly fewer filopodia than each single alone, but an additive effect of the two mutations combined could not be excluded (Figure 6B). Some *arx-7(ok1118M+); unc-34(e951) *double mutants lacked a strong posterior neurite from PQR (2 of 17), similar to *arx; unc-115 *double mutants, suggesting that UNC-34/Enabled and the Arp2/3 complex might control the ability of a neurite to form or to extend. *unc-34 *mutants did not display a reduction in PQR growth cone perimeter (7.78 ± 0.9 μm compared to 7.96 ± 0.91 μm for wild type; *P *= 0.387; Figure 6C).

These data indicate that Arp2/3 and UNC-115 affect both growth cone filopodia and growth cone perimeter, which might reflect lamellipodial protrusion. In contrast, *unc-34 *affected only growth cone filopodia but not growth cone perimeter. Thus, growth cone perimeter and number of filopodia are at least partially independent processes. For example, *unc-115 *alone affected growth cone perimeter but not filopodia, and *unc-34 *affected filopodia but not growth cone size. These results indicate that the filopodial defects seen in *arx *mutants might be a specific defect in filopodia and are unlikely to simply be a secondary effect of reduced lamellipodial protrusion.

### ARX-7, UNC-115/abLIM, and UNC-34 affect filopodia initiation but not duration or length

The effects of Arp2/3, UNC-115, and UNC-34 on PQR growth cone filopodia formation could be due to decreased filopodial stability or half-life, or could be due to a decrease in the rate of filopodial initiation. We imaged growth cones from wild type and mutants over time to assess the dynamic effects of these mutations of filopodia formation. The commissural growth cones of the VD motor axons have been used in previous time-lapse studies [31,42]. The VD axons begin extension during the L1/L2 larval molt and continue to extend early in the second larval stage. As the VD commissural growth cones emerge dorsally after traversing the ventral muscle quadrant, they display a spread appearance with multiple dynamic filopodial extensions [42]. Until the growth cones reach the dorsal muscle quadrant, they generally retain a spread morphology with multiple filopodial extensions ([42] and our observations).

**Figure 8:**
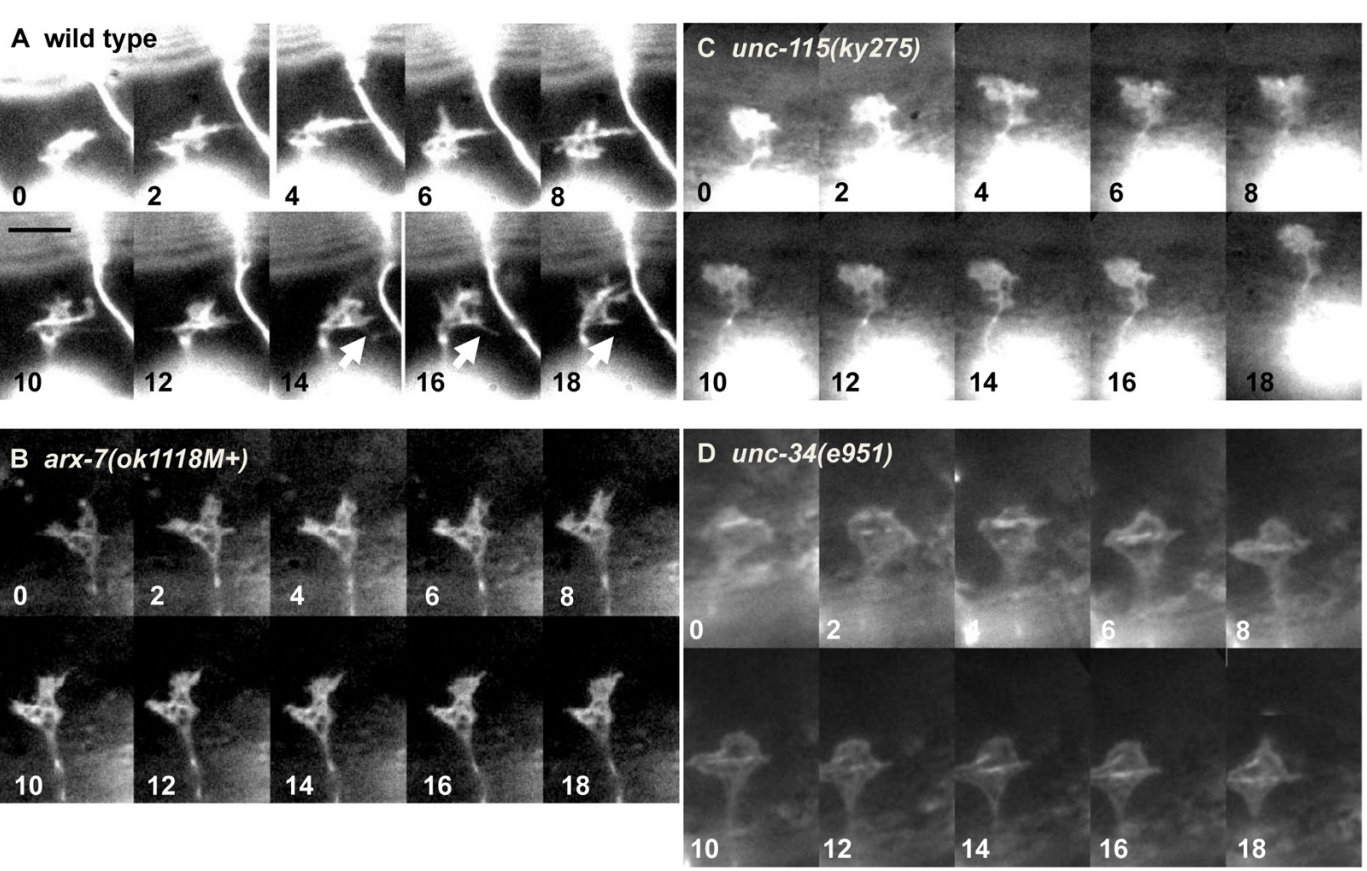
**Time lapse images of VD growth cone outgrowth**. Series of time-lapse images of VD growth cones in early L2 larvae as they migrate between muscle quadrants. The time in minutes after initiation of the experiment are shown in the lower left corner of each image. The scale bar represents 5 μm. **(A) **A wild-type VD growth cone displays multiple protrusions. The arrow points to the initiation (at 14 minutes) and retraction (at 18 minutes) of a growth cone filopodium. **(B) **An *arx-7(ok1118M+) *growth cone displayed fewer filopodial protrusions and generally less dynamics than wild type. **(C) **An *unc-115(ky275) *growth cone displayed few filopodia and generally reduced dynamics. **(D) **An *unc-34(e951) *growth cone as it spread along a nerve tract as described in [42]. The growth cone displayed few filopodial protrusions.

To understand the roles of Arp2/3 and UNC-115 in filopodia formation, we imaged VD growth cones as they migrated between the ventral and dorsal muscle quadrants. In wild type, the VD growth cones displayed dynamic extension and retraction of lamellipodia-like and filopodia-like projections (Figure 8A; Additional file 1). In *arx-7*, *unc-115 *and *unc-34 *mutants, VD growth cone dynamics appeared generally reduced, and the growth cones displayed a less ramified appearance (Figure 8B-D; Additional files 2, 3 and 4). Notably, fewer filopodia were observed on the growth cones of mutants, consistent with results in PQR.

**Figure 9:**
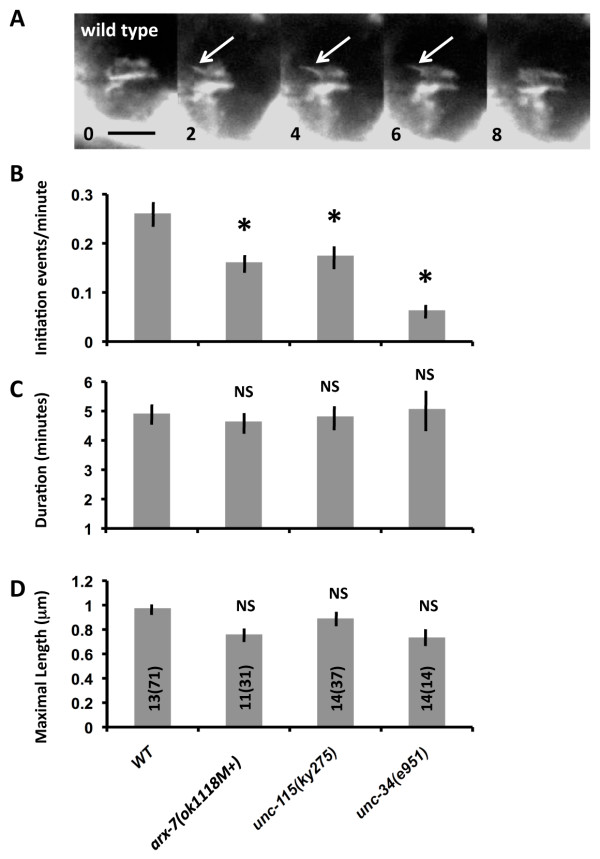
**Quantification of VD/DD growth cone dynamics**. **(A) **A time lapse series of a wild-type VD growth cone showing the emergence and retraction of an individual filopodium (arrows). The scale bar represents 5 μm, and the time after the start of the time lapse experiment is indicated in the lower left of each panel. **(B-D) **Quantification of filopodia initiation rate, stability, and maximal length. Error bars represent standard error of the mean. Asterisks indicate significant differences between wild type and the mutant (*P *< 0.0006). NS = not significantly different. The number of growth cones analyzed and the total number of filopodia (in parentheses) is indicated in the bars in (D). (B) Initiation rates of filopodia (average initiation events per minute); (C) average filopodial duration (in minutes); and (D) average filopodial maximal length.

Reduced steady-state filopodia number could be due to a defect in filopodia initiation or filopodia stability or duration. We tracked the emergence and dynamics of multiple individual filopodial from multiple growth cones over time (Figure 9A). This analysis revealed that filopodia had an initiation rate of 0.26 filopodia per minute in wild type (approximately one new filopodium initiated every 4 minutes; Figure 9B). Filopodia initiation rate was significantly reduced in *arx-7*, *unc-115*, and *unc-34 *mutants (Figure 9B): 0.16 per minute, 0.17 per minute, and 0.07 per minute, respectively. Filopodia did occasionally form in these mutants, and we measured their duration time and maximal length. We found no significant difference between wild type and any of these mutants in filopodial duration (4 to 5.5 minutes; Figure 9C) or maximal filopodial length (0.8 to 1 μm; Figure 9D). These data indicate that *arx-7*, *unc-115*, and *unc-34 *primarily affected the rate of filopodia initiation but did not affect the duration or maximal length of filopodia once they had formed. However, we cannot rule out the possibility that some filopodia initiate but abort before they are detectable in our assay.

We also determined the average translocation rates of wild-type and mutant growth cones (see Materials and methods; Table 1). Wild type had a translocation rate of 7.38 μm/h. This rate is consistent with what has been observed previously for VD growth cones in mounted and anaesthetized larvae (4 to 8.7 μm/h) [42]. *arx-7 *and *unc-34 *had significantly reduced growth cone translocation rates: 4.08 μm/h for *arx-7 *and 3.84 μm/h for *unc-34 *(Table 1). *unc-115 *did not have a significantly reduced translocation rate (7.02 μm/h). Variability in translocation rate in each genotype, including wild type, was very high, making it difficult to assign a direct effect on growth cone morphology to translocation rate. For example, *unc-115 *affected filopodia to the same extent as *arx-7 *but did not affect translocation rate. It is possible, however, that these mutants have distinct effects on growth cones aside from filopodia that were not assayed in these studies.

**Table 1:** Growth cone translocation rates in cytoskeletal mutants.

**Genotype (n)**	**Translocation rate** **(μm/h)**	***t*-test *P*-value versus wild-type**
Wild type (6)	7.38 ± 1.86	NA
*arx-7(ok1118M+) *(6)	4.08 ± 1.74	0.009
*unc-115(ky275) *(6)	7.02 ± 1.32	0.700
*unc-34(e951) *(6)	3.84 ± 2.88	0.034

NA, not applicable.

**Figure 10:**
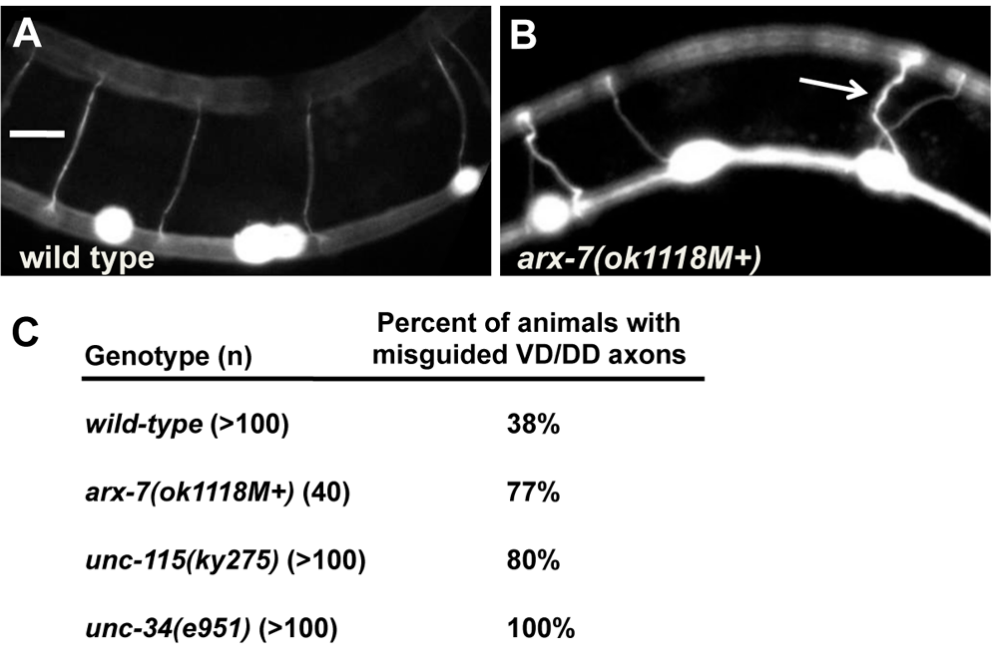
**VD/DD axon guidance defects**. **(A, B) **Micrographs of young adult animals with *unc-25::gfp *expression in the VD/DD motor axons. In wild type, the commissural axons generally extend directly from ventral to dorsal. In *arx-7(ok1118M+)*, axons often wandered laterally and crossed other commissures before reaching the dorsal cord (arrow). **(C) **Percentage of animals with misguided VD/DD commissural axons.

*unc-115 *and *unc-34 *have been shown previously to have VD/DD axon guidance defects [14,16,18]. *arx-7 *mutants also displayed VD/DD pathfinding defects (compare Figure 10A and 10B). In *arx-7*, VD and DD axons wandered laterally and crossed other commissural axons in 77% (n = 40) of animals (Figure 10C). All *arx-7 *VD/DD axons reached the dorsal nerve cord. Such crossings were occasionally observed in wild type (38%, n = 100), but occurred at a significantly higher rate in *arx-7 *(*P *< 0.0001). While the penetrance of axon guidance defects of *arx-7*, *unc-115*, and *unc-34 *were similar (Figure 10C), the misguided axons in *arx-7 *always reached the dorsal cord, whereas misguided *unc-115 *and *unc-34 *axons wandered laterally, terminated prematurely, and sometimes failed to reach the dorsal nerve cord [14,16]. The filopodia defects of *arx-7 *were as severe as those of *unc-34 *and *unc-115*, suggesting that *unc-115 *and *unc-34 *might have other, non-filopodial roles in axon guidance not shared by *arx-7*.

## Discussion

### The Arp2/3 complex controls growth cone size and filopodia formation *in vivo*

The results presented here show that the Arp2/3 complex is required for the initiation of growth cone filopodia of *C. elegans *neurons *in vivo*. The role of Arp2/3 in growth cone morphology has been controversial, based on studies in cultured neurons. Some studies concluded that Arp2/3 was not involved in growth cone filopodia formation [11,12], while another demonstrated a role for Arp2/3 in growth cone filopodia formation [13]. Our results presented here clearly demonstrate that Arp2/3 is required for growth cone filopodia formation *in vivo*. The previous study that found no role of Arp2/3 in cultured hippocampal growth cone filopodia formation used expression of a dominant negative WAVE construct to inhibit Arp2/3 [11], whereas the study that found an effect of Arp2/3 in hippocampal growth cones used small interfering RNA knockdown of the p34-Arc subunit of the Arp2/3 complex [13]. Possibly, the dominant-negative WAVE construct did not inhibit Arp2/3 as well as the small interfering RNA. In any case, our studies of *C. elegans *growth cones *in vivo *demonstrate that Arp2/3 is a key regulator of growth cone filopodia formation.

A previous study using cultured hippocampal neurons [13] showed that Arp2/3 was present in the growth cone at branch points of actin filaments as observed in other cell types, and filaments with Arp2/3 at their base contributed to filopodial bundles, suggesting that Arp2/3 provides a source of actin filaments for filopodial formation [13]. The nature of the growth cone cytoskeletal defects in *arx *mutant growth cones is difficult to discern, as the growth cones are very small compared to those of cultured hippocampal neurons (<5 μm in diameter compared to approximately 20 μm for cultured hippocampal neurons). However, it is possible that there is reduced nucleation of lamellipodial actin filaments in *arx-7 *mutant growth cones, resulting in less availability of lamellipodial actin filaments to contribute to filopodial bundles. This idea is consistent with the time-lapse analysis of growth cone filopodia, which showed that *arx *mutants had reduced filopodial initiation rates compared to wild type (Figure 9). *arx-7 *mutant PQR growth cones also displayed a reduction in the size of the growth cone (Figures 5 and 6), consistent with a role of Arp2/3 in the nucleation of growth cone lamellipodial actin filaments. Interestingly, once filopodia formed in *arx-*7 mutants, they had characteristics similar to wild type, including time of persistence/stability and maximal length. Thus, the Arp2/3 complex might primarily affect the initiation of filopodia and have only a minor role in filopodial stability or extension once a filopodium has initiated. However, we cannot rule out the possibility that filopodia initiate and collapse before they are visible as filopodia in our imaging assays.

### The Arp2/3 complex is required for axon guidance

As a growth cone navigates its environment, filopodia are thought to act as sensors of extracellular cues and have a role in guiding the growth cone [43,44]. Consistent with this idea, we found that *arx *mutants have defects in the guidance of the PDE and VD/DD axons and of the PQR dendrite. Reduction of filopodia on *arx *mutant growth cones might lead to growth cone guidance defects. However, *arx *axon guidance defects were incompletely penetrant (for example, 15 to 34% for the PDEs). This could be due to the action of the remaining few filopodia on *arx *mutant growth cones or could be due to lamellipodial guidance. Indeed, *unc-34/Enabled *mutants lack most or all growth cone filopodia but have relatively weak effects on axon guidance ([16,18,40] and this work).

**Figure 11:**
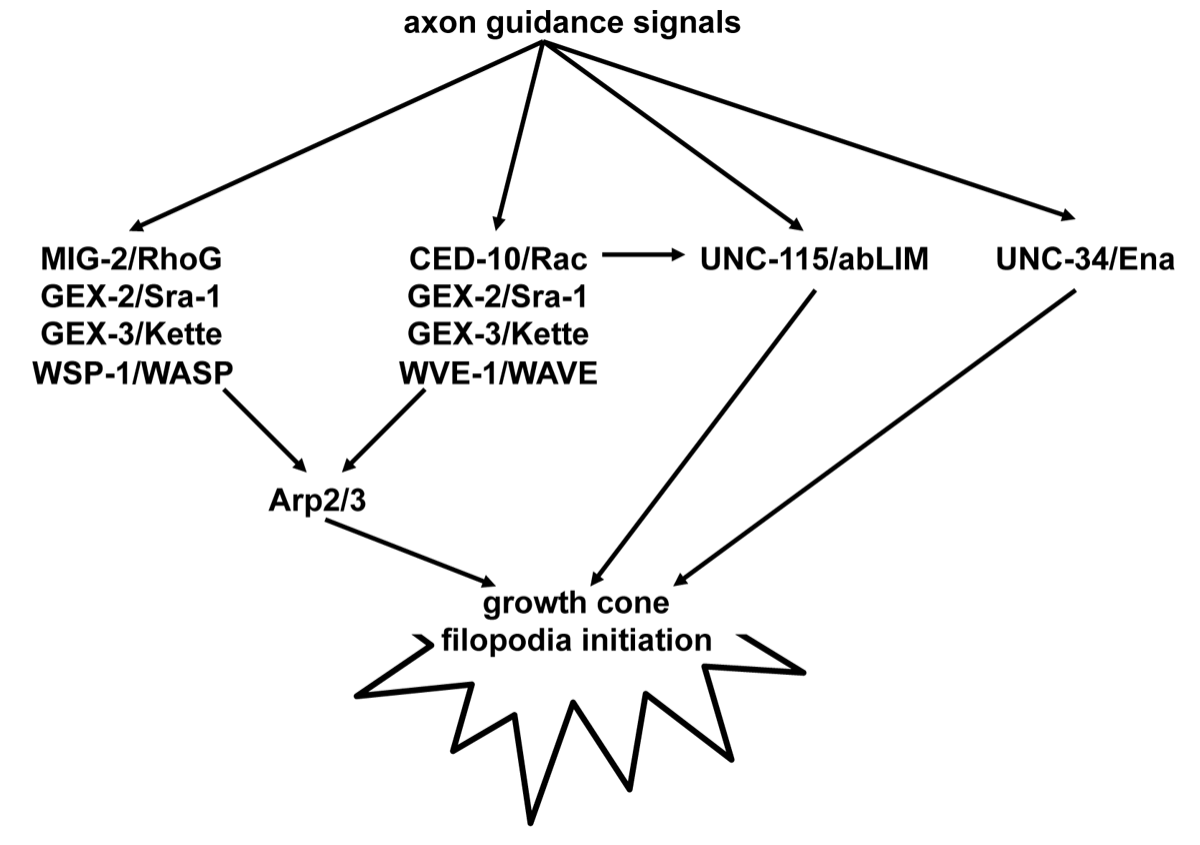
**A model of Arp2/3, UNC-115/abLIM, and UNC-34 in growth cone filopodia formation**. Arp2/3 might act downstream of both CED-10/Rac and MIG-2/RhoG and in parallel to UNC-115/abLIM and UNC-34/Enabled. CED-10/Rac might also regulate UNC-115/abLIM.

Rac GTPases are thought to regulate Arp2/3 activity via the WAVE family of Arp2/3 activators: WAVE is present in a complex with Sra-1 and kette, and Rac-GTP binding to Sra-1 activates WAVE and thus Arp2/3 [45,46]. Previous studies showed that in *C. elegans *PDE axon guidance, CED-10 Rac acts with WVE-1/WAVE, and that MIG-2/RhoG acts with the WSP-1/WASP molecule, a member of the WASP family of Arp2/3 activators [10]. Shown here is that PDE axon guidance defects of *arx *mutations were enhanced by both *ced-10 *and *mig-2*, consistent with a role of both CED-10/Rac and MIG-2/RhoG in the Arp2/3 pathway (Figure 11). CED-10/Rac might also control UNC-115/abLIM [15], explaining the enhancement of *arx *mutations by *ced-10*.

### UNC-115/abLIM controls growth cone size and filopodia formation

As mentioned above, the weak axon guidance defects of *arx *mutants could be explained by the lamellipodial guidance or by the activity of the remaining few filopodia on mutant growth cones, presumably formed by Arp2/3-independent mechanisms. UNC-115/abLIM has previously been implicated in axon pathfinding, but the role of the molecule in regulation of growth cone dynamics and morphology during outgrowth had not been demonstrated. We found that UNC-115/abLIM also affected the formation of growth cone filopodia. While *unc-115 *displayed no significant reduction of filopodia on PQR growth cones, it did enhance filopodia defects of *arx-7*. Furthermore, *unc-115 *displayed reduced filopodia initiation rate on VD/DD growth cones. These data indicate that UNC-115/abLIM is also a regulator of filopodia initiation similar to Arp2/3. That *unc-115/abLIM *mutants alone have defects in VD filopodia but not PQR filopodia suggests that different neurons have distinct requirements for UNC-115/abLIM in growth cone filopodia formation. This could be due to different guidance signals detected by these neurons that trigger distinct downstream signal transduction pathways, resulting in different requirements for UNC-115/abLIM.

UNC-115/abLIM binds to actin filaments via the carboxy-terminal villin headpiece domain (VHD), and promotes the formation of lamellipodia and filopodia in *C. elegans *neurons and cultured fibroblasts [15,23]. UNC-115/abLIM might act as a bundling protein in filopodial bundles, or it might produce new actin filaments for use in filopodia, similar to Arp2/3. Indeed, the villin headpiece domain from human villin can nucleate actin polymerization [47], but this activity has not been described for UNC-115/abLIM. Our results here suggest that UNC-115/abLIM might also have a role in lamellipodia organization similar to Arp2/3, as *unc-115 *mutants also displayed reduced PQR growth cone size.

### UNC-34/Enabled affects filopodia formation without affecting growth cone morphology

Enabled has been extensively implicated in filopodia formation, including on growth cones, and our results that *unc-34 *mutant growth cones had reduced filopodia numbers confirm this role of Enabled. In time-lapse studies, we found that *unc-34 *affected the rate of filopodia formation, suggesting that Enabled controls filopodia initiation similar to Arp2/3 and UNC-115/abLIM. This is similar to previous results in cultured neurons in response to netrin [19]. We found that *unc-34 *had no effect on filopodia stability or length once a rare filopodium formed, indicating that UNC-34 has no role in filopodia outgrowth or stability. This is in contrast to cultured neurons, where Ena/VASP inhibition also resulted in filopodia extension defects [19].

In contrast to *arx-7 *and *unc-115 *mutants, *unc-34 *mutant PQR growth cones, while nearly devoid of filopodia, displayed no significant reduction in size. While all three mutations caused reduced filopodia initiation, this difference might hint at distinct mechanisms of action of these molecules. Possibly, Arp2/3 and UNC-115/abLIM primarily affect lamellipodial actin organization such that filaments are less available for filopodia formation in mutants. In contrast, UNC-34/Enabled had a filopodia-specific effect, consistent with the role of Ena/VASP molecules as actin anti-capping proteins. It is surprising that *unc-34 *had little or no effect on stability or length of the few filopodia that did form in these mutants. Possibly, the anti-capping activity of UNC-34/Enabled is required only during initiation and other proteins can serve its function during elongation. It is also possible that filopodial initiation requires other actin-interacting properties of Enabled that have been described recently (for example, filament nucleation or bundling) [21,22].

### Arp2/3, UNC-115/abLIM, and UNC-34/Enabled act together in axon guidance

Our results indicate that Arp2/3, UNC-115/abLIM, and UNC-34/Enabled are required for growth cone filopodia formation. However, the effects of these mutations on PDE and VD/DD axon guidance and PQR dendritic guidance were incompletely penetrant. Strikingly, double mutants of *arx *with *unc-115 *and *unc-34 *displayed greatly enhanced PDE axon guidance defects, and previous studies showed that *unc-34 *and *unc-115 *had overlapping roles in PDE axon guidance. Possibly, perturbing multiple actin modulating molecules had general, nonspecific effects on the cytoskeleton, resulting in increased defects. We think this is not the case as these interactions were specific. *erm-1 *encodes the only member of the ezrin/radixin/moesin family of actin-membrane linking proteins in the *C. elegans *genome. *erm-1 *mutation caused weak guidance defects on its own, indicating that ERM-1 is required for axon guidance. However, *erm-1 *did not enhance *unc-115 *or *unc-34*, suggesting that the enhancement of *arx*, *unc-115*, and *unc-34 *is specific to these genes and is not the result of general cytoskeletal disruption.

Together, our data indicate that Arp2/3, UNC-115/abLIM, and UNC34/Enabled might act in a common process in axon guidance, namely in the initiation of growth cone filopodia (Figure 11). Indeed, *unc-115 *and *unc-34 *enhanced the PQR filopodia defects of *arx-7*, although the enhancement by *unc-34 *might simply be an additive effect because *unc-34 *mutants alone display severely reduced filopodia numbers. It is also possible that all three molecules affect filopodia formation and have redundant roles in other aspects of growth cone morphology. Indeed, this seems to be the case, as both *unc-115; arx *and *unc-34; arx *double mutants displayed severe PQR neurite outgrowth and formation defects. This is surprising for *unc-34/Enabled*, as Ena/VASP molecules were traditionally considered to be anti-capping proteins with specific effects on filopodia. However, recent studies have suggested that Ena/VASP molecules have other effects on actin, including bundling and nucleation [21], have anti-capping-independent roles in filopodia formation [22] and control the initiation of neurites in the mammalian central nervous system [48,49]. Possibly these other roles of UNC-34/Enabled explain the severe defects in neurite formation in *unc-34; arx *double mutants.

## Conclusion

Many molecules have been implicated in axon guidance through genetic, molecular, and biochemical studies. The effects of these molecules on the behavior and dynamics of the growth cone during axon guidance are unclear in many cases. Through a combination of genetic analysis and time-lapse imaging of growth cones during outgrowth, we show that the Arp2/3 complex acts cell autonomously in the initiation of new growth cone filopodia during growth cone outgrowth, possibly explaining the axon guidance defects observed in *arx *mutants. We show that two other actin modulatory molecules, UNC-115/abLIM and UNC-34/Enabled, also affect growth cone filopodia initiation, which might explain the genetic redundancy of Arp2/3, UNC-115/abLIM, and UNC-34/Enabled in axon guidance. Arp2/3 and UNC-115/abLIM, but not UNC-34/Enabled, also affect the size of the growth cone, possibly reflecting a role in the formation of growth cone lamellipodium. Thus, multiple actin regulatory molecules have shared roles (filopodia initiation) and distinct roles (lamellipodia formation) in the growth cone during axon outgrowth.

## Abbreviations

ERM: Ezrin/radixin/moesin; GFP: green fluorescent protein; Pvl: protruding vulva; VNC: ventral nerve cord.

## Competing interests

The authors declare that they have no competing interests.

## Authors' contributions

ADN, JOC, and EAL conceived of and designed the experiments, EAL and ADN performed the experiments, and JOC performed the cell-autonomous rescue of *arx-4*. EAL, with help from ADN and JOC, wrote the paper.

## Supplementary Material

Additional file 1 **Time-lapse movie of a wild-type VD growth cone migrating between ventral and dorsal muscle quadrants with typical dynamic appearance**. Images captured every 120 s, total movie duration 36 minutes. The scale bar in the first frame represents 5 μm. Click here for file

Additional file 2 **Time-lapse movie of an *arx-7(ok1118M+) *growth cone**. Images captured every 120 s, total movie duration 30 minutes. The scale bar in the first frame represents 5 μm. Click here for file

Additional file 3 **Time-lapse movie of an *unc-115(ky275) *growth cone**. Images captured every 120 s, total movie duration 24 minutes. The scale bar in the first frame represents 5 μm. Click here for file

Additional file 4 **Time-lapse movie of *unc-34(e951) *growth cone**. Images captured every 120 s, total movie duration 54 minutes. The scale bar in the first frame represents 5 μm. Click here for file
